# Automated Self-Administered 24-H Dietary Assessment Tool (ASA24) recalls for parent proxy-reporting of children’s intake (> 4 years of age): a feasibility study

**DOI:** 10.1186/s40814-021-00864-6

**Published:** 2021-06-11

**Authors:** Isobel Sharpe, Sharon I. Kirkpatrick, Brendan T. Smith, Charles D. G. Keown-Stoneman, Jessica Omand, Shelley Vanderhout, Jonathon L. Maguire, Catherine S. Birken, Laura N. Anderson, on behalf of the TARGet Kids! collaboration

**Affiliations:** 1 Department of Health Research Methods, Evidence, and Impact, McMaster University, Hamilton, Ontario Canada; 2 School of Public Health and Health Systems, University of Waterloo, Waterloo, Ontario Canada; 3 Department of Health Promotion, Chronic Disease and Injury Prevention, Public Health Ontario, Toronto, Ontario Canada; 4 Division of Epidemiology, Dalla Lana School of Public Health, University of Toronto, Toronto, Ontario Canada; 5 Applied Health Research Centre of the Li Ka Shing Knowledge Institute of St. Michael’s Hospital, University of Toronto, Toronto, Ontario Canada; 6 Division of Biostatistics, Dalla Lana School of Public Health, University of Toronto, Toronto, Ontario Canada; 7 Division of Child Health Evaluative Sciences (CHES), Sick Kids Research Institute, Toronto, Ontario Canada; 8 Department of Nutritional Sciences, Faculty of Medicine, University of Toronto, Toronto, Ontario Canada; 9 Department of Pediatrics, St. Michael’s Hospital, Toronto, Ontario Canada

**Keywords:** Measurement, Child, Parents, Self-report, Nutrition assessment, Nutrition surveys

## Abstract

**Background:**

Robust measurement of dietary intake in population studies of children is critical to better understand the diet–health nexus. It is unknown whether parent proxy-report of children’s dietary intake through online 24-h recalls is feasible in large cohort studies.

**Objectives:**

The primary objective of this study was to describe the feasibility of the Automated Self-Administered 24-h Dietary Assessment Tool (ASA24) to measure parent proxy-reported child dietary intake. A secondary objective was to compare intake estimates with those from national surveillance.

**Methods:**

Parents of children aged 4–15 years participating in the TARGet Kids! research network in Toronto, Canada were invited by email to complete an online ASA24-Canada-2016 recall for their child, with a subsample prompted to complete a second recall about 2 weeks later. Descriptive statistics were reported for ASA24 completion characteristics and intake of several nutrients. Comparisons were made to the 2015 Canadian Community Health Survey (CCHS) 24-h recall data.

**Results:**

A total of 163 parents completed the first recall, and 46 completed the second, reflecting response rates of 35% and 59%, respectively. Seven (4%) first recalls and one (2%) second recall were excluded for ineligibility, missing data, or inadvertent parental self-report. The median number of foods reported on the first recall was 18.0 (interquartile range (IQR) 6.0) and median time to complete was 29.5 min (IQR 17.0). Nutrient intakes for energy, total fat, protein, carbohydrates, fiber, sodium, total sugars, and added sugars were similar across the two recalls and the CCHS.

**Conclusions:**

The ASA24 was found to be feasible for parent proxy-reporting of children’s intake and to yield intake estimates comparable to those from national surveillance, but strategies are needed to increase response rate and support completion to enhance generalizability.

**Supplementary Information:**

The online version contains supplementary material available at 10.1186/s40814-021-00864-6.

## Key messages regarding feasibility

- *What uncertainties existed regarding the feasibility?* The Automated Self-Administered 24-H Dietary Assessment Tool (ASA24) is a self-reported 24-h recall developed by the US National Cancer Institute. With the convenience of an online system, it has broad applications for collecting nutritional information within epidemiological cohorts; however, there are limited reports of its feasibility as a tool for parent report of child intake. Previous studies have found the ASA24 to be feasible among adults and adolescents; however, little research has focused on younger children. The feasibility considerations for young children, especially those under 10 years of age, are unique given that parent proxy-reporting is often used.
- *What are the key feasibility findings?* The response rate for the ASA24 recall was 35%. Of those completers who were asked to perform a second recall, the response rate was 59%. Few responses (4% of first recalls) were excluded from the analysis for ineligibility, missing data, or inadvertent parental self-report. The median amount of time to complete the first recall was 29.5 min (interquartile range 17.0 min). Further, nutrient intakes for energy, total fat, protein, carbohydrates, fiber, sodium, total sugars, and added sugars were similar between the ASA24 and an interview-administered 24-h recall from the Canadian Community Health Survey.
- *What are the implications of the feasibility findings for the design of the main study?* The findings from our study suggest that the ASA24 is an overall feasible approach for collecting parent proxy-reported recall data among young children. This will create opportunities for its use as a dietary assessment tool within larger cohort studies aiming to understand the relationship between dietary factors and long-term health outcomes.

## Background

Measuring dietary intake in childhood is important for evaluating population health, as well as for assessing associations between patterns of eating and health outcomes [1, 2]. Childhood eating patterns may track into later life [3, 4], with implications for health and disease risk across the life course. For instance, poor eating patterns in early life and childhood has been associated with adulthood cardiovascular disease [5]. Furthermore, the 2016 Global Burden of Diseases, Injuries, and Risk Factors Study found that in Canada, dietary risk was the greatest attributable factor to death, representing 17.6% of total deaths [6]. Consequently, the accurate measurement of childhood dietary intake is necessary for informing guidelines and interventions to improve health.

Self-reported measures are commonly used to quantify dietary intake in nutritional epidemiology [7]. These measures are prone to measurement error from a range of sources, including imperfect memory [8]. However, as compared with objective measures, which are relatively rare in the field of dietary assessment as well as burdensome and expensive, self-reported tools provide the ability to collect data on a wide range of foods and drinks and offer relative ease of administration [7, 9]. Food frequency questionnaires (FFQs) have often been used in epidemiologic studies due to their low cost, but validation research suggests that 24-h recalls capture intake with less systematic error [10, 11].

The completion of 24-h recalls involves prompting respondents to report all foods and drinks consumed either over the past 24 h or over the previous calendar day, and traditionally have been conducted by highly trained interviewers, with trained coders needed to link the data to food composition databases [12]. In recent years, innovative tools have been developed to improve the feasibility of collecting recalls in large-scale research, such as epidemiologic cohorts. One such tool is the Automated Self-Administered 24-H Dietary Assessment Tool (ASA24), which was developed by the US National Cancer Institute based on the multiple-pass method used in national surveillance [13], including the Canadian Community Health Survey (CCHS) [14]. The ASA24 system is freely available for use by researchers and has been adapted specifically for use in Canada, with adaptations to the Canadian food supply and linkages to the Canadian Nutrient File [15].

The literature shows that ASA24 recalls are feasible and perform well relative to true intake and interviewer-administered recalls in samples of adults [16, 17]. Over recent years, several studies have assessed the feasibility of its use among older children, namely from ages 10 to 13 years [15, 18–22]. However, there has been relatively little research to inform the feasibility of ASA24 to assess dietary intake in young children. Specific considerations related to collecting intake data for children include their cognitive stage, literacy and numeracy skills, and knowledge of foods and their preparation [23–25]. Parents or other proxy reporters are often called upon to report young children’s intake, with 10 years suggested as the age at which children may be able to report independently [25]. One small validation feeding study with preschoolers found that parents were able to report their children’s intake for a single day relatively well using ASA24 [26], but the results do not provide insights into the feasibility of ASA24 for use in community-based samples.

The objective of this study was thus to examine the feasibility of use of ASA24-Canada-2016 recalls to measure parent proxy-reported dietary intake among children four years of age and older. The secondary objective was to compare intake estimates from ASA24 with those yielded by national surveillance data collected using interviewer-administered multiple pass 24-h recalls.

## Methods

### Study design and respondents

A feasibility study was conducted between March 3rd, 2018 and June 4th, 2019 to describe the use of ASA24-Canada-2016 among children participating in The Applied Research Group for Kids (TARGet Kids!) cohort study. TARGet Kids! is a primary care practice-based research network for children primarily in the Greater Toronto Area, Ontario, Canada [27]. On an ongoing basis, children under six years of age are recruited into TARGet Kids! from primary care practices (pediatrics or family medicine) by trained research assistants and followed prospectively throughout childhood and adolescence at annual well-child visits. Children were excluded at enrollment if they were < 32 weeks gestational age, had non-English-speaking parents, had growth-restricting health conditions such as cystic fibrosis or failure to thrive, severe developmental delay, or other chronic conditions (not including asthma and high-functioning autism). Over 10,500 children have been recruited into TARGet Kids! since its inception in 2008. For this feasibility study, children were included if they completed a TARGet Kids! visit (either baseline or follow-up) between March 2018 and June 2019 and were at least 4 years of age at the time of the visit. This lower age cutoff was chosen to avoid overburdening the parents of younger children in the TARGet Kids! cohort who are asked to complete several other age-specific questionnaires. No upper age limit was applied.

### Data collection

Parents were sent a standardized email by a trained research assistant requesting online completion of ASA24-Canada-2016 for their child. The email included a link to the ASA24 and a username and password. Parents completed ASA24 independently at home (or their choice of location). When parents logged in to ASA24, they were asked to report all foods and drinks consumed within the previous 24 h from the time of completion. Within the email, parents were requested to “*remember to complete it only for your child’s dietary intake (not for yourself)*”, since ASA24 queries respondents about their own intake and does not offer an option to change prompts to facilitate proxy-reporting. Parents were asked to complete ASA24 for either the day the email was sent or the day after such that the recall was at least somewhat unannounced, which is recommended to reduce the likelihood that parents will alter their children’s eating patterns due to social desirability bias [28]. If the recall was not completed, the research assistant sent one reminder email after 1 week, and a second reminder after 2 weeks.

Since recalls capture intake for a given day, large surveys often collect a second non-consecutive recall from a subsample to allow modeling of distributions of usual intake, which entails partitioning within- and between-person variation and removing the within-person variation (which mostly comes from day-to-day variation) such that inferences can be made about proportions above and below recommendations, for example [29]. Thus, approximately 25% of respondents who completed the first recall were asked to complete a second recall about 2 weeks later, with reminder emails again sent after 1 week and after 2 weeks to respondents who had not yet completed.

When the feasibility study began in March 2018, no incentive was provided to parents; however, due to low response rate, an incentive was introduced on November 28th, 2018 to encourage completion. Parents were informed in the email asking them to complete ASA24 that they would receive a $5 electronic gift card (for a coffee shop) for both the first and second recall. During the 16-month period of this feasibility study, 8 months of data collection were conducted with no incentive, and the subsequent 8 months were conducted with the incentive.

In addition to the interface used by respondents to complete the recall, ASA24 includes a researcher website from which recall data are downloaded. These data were linked by study identification number to survey and anthropometric data from the most recent TARGet Kids! visit. Parent-completed survey data included child age and sex, maternal ethnicity, and family income. Child height and weight were measured by trained research assistants, and body mass index (BMI) z scores were calculated using the recommended World Health Organization Standards and Reference [30].

Parents provided written consent for participation in TARGet Kids!. Research ethics board approval was obtained from the Hospital for Sick Children, St. Michael’s Hospital, Toronto, Ontario and the Hamilton Research Ethics Board, Hamilton, Ontario.

### Data cleaning

Each food and beverage reported by respondents was automatically coded within ASA24 based on a database adapted from the Canadian Nutrient File [31]. ASA24 captured portion size by prompting respondents to choose between various images of the food items. For each respondent, details of each individual food and beverage item reported as well as nutrient intake for macro- and micronutrients for each item were downloaded from the ASA24 researcher website. As a note, the ASA24 does not capture discretionary salt use given that most dietary salt intake comes from processed foods. Guidelines from the US National Cancer Institute were applied for data cleaning [32]. Reports identified as breakoffs (i.e., the participant entered some food items but exited ASA24 before reaching the final question) were removed due to missing data. The data were manually scanned for open-ended text entries that required review or duplicate food item entries, but none were identified. Additionally, entries considered to be implausible for children, such as wine, espresso, > 1 g of alcohol, or > 50 g of caffeine were manually inspected to evaluate whether intake was inadvertently recalled for the parent instead of the child. This inspection included an assessment of the child’s age, as some older children in the sample may consume alcohol or caffeine themselves, but all of the children identified with these alcohol and caffeine values were younger (< 9 years of age), and thus these responses were excluded.

### Statistical analysis

The examination of feasibility focused on response rates for each recall, number of attempts (i.e., logins to the online system) needed before the recall was completed, number of foods entered, and time to complete the recall. The response rate for each recall was compared pre- and post-introduction of the incentive. Further, we examined whether the recall was indicative of typical dietary intake based on the final ASA24 question, which asks participants if their intake that day was “much less than usual intake”, “usual intake”, or “much more than usual intake”. Descriptive statistics were calculated as the mean and standard deviation (SD) or the median and interquartile range (IQR) for continuous measures and as frequencies (%) for categorical measures. Descriptive statistics were used to characterize the study population, including child age, sex, and number of siblings, maternal ethnicity, family income, and BMI z score. The demographic characteristics of ASA24 non-respondents compared with those of the respondents.

Descriptive statistics, including means (95% confidence interval (CI)), and medians (IQR), were calculated for eight nutrients: energy, total fat, protein, carbohydrates, fiber, sodium, total sugars, and added sugars. These estimates were compared with estimates from the CCHS 2015-Nutrition share file [33] using the 95% CIs. The 2015 CCHS included 20,487 Canadians over 1 year of age and collected intake data by interviewer-administered 24-h recalls completed using the same multiple-pass method that informed ASA24 [13, 14]. For the purposes of estimating mean and median intakes, the first recall was used (second recalls available for a subsample were not used in the present analyses). Data from children aged 4 to 15 years (n = 4124) were included to match the age range of the TARGet Kids! respondents for whom ASA24 data were available. Statistics Canada’s survey weights were applied to the CCHS analyses and confidence intervals were estimated using bootstrapping with 500 balanced repeated replications to account for the complex sampling design [14]. All statistical analyses were performed using SAS Studio 3.71 (SAS Institute Inc., Cary, NC, USA).

## Results

**Fig. 1:**
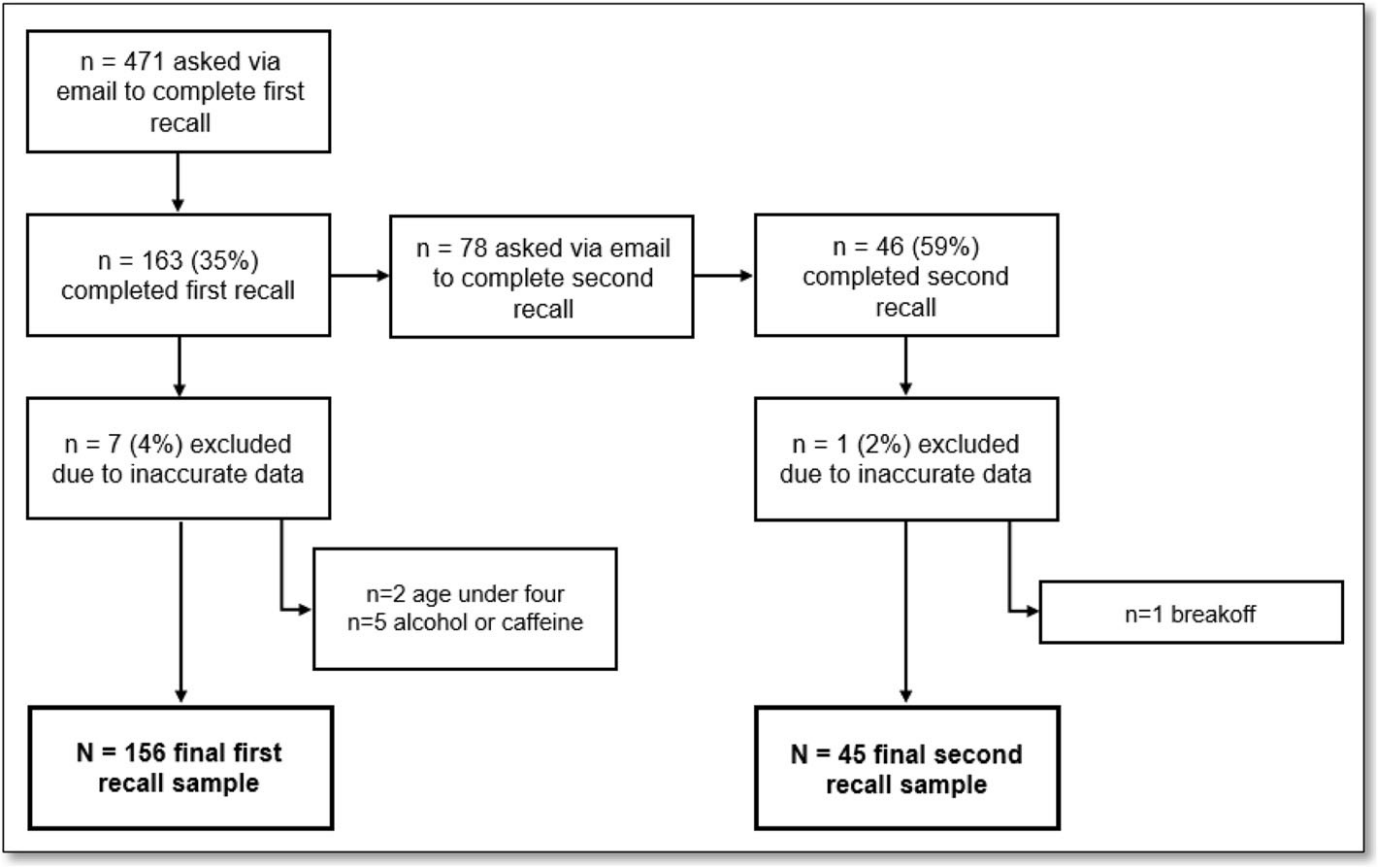
Flow chart of respondents throughout study, showing reasons for exclusion and the final sample sizes for both the first and second recalls

Figure 1 outlines the flow of respondents throughout the study. During the study period, 471 parents with children in the TARGet Kids! cohort were invited to complete ASA24. A total of 163 (35%) parents contacted by email completed a first recall for their children. Seven (4%) recalls completed by these respondents were excluded because children were found to not meet the eligibility criteria (below 4 years of age; n = 2), or parents were suspected to have reported their own rather than the children’s intake (n = 5), leaving 156 children with data for the first recall. Four recalls included five or fewer foods, but the parents indicated that the reported foods and beverages represented usual intake for their children; the data from these recalls were therefore retained. There were 308 non-respondents. Out of these, 301 were included in the non-respondent analysis; seven (2%) were removed for not meeting the study eligibility criteria (below 4 years of age).

A total of 78 parents who completed the first recall were asked to complete a second recall. Forty-six (59%) second recalls were completed and one (2%) was excluded as a breakoff, leaving 45 children with data from a second recall. None of the second recalls appeared incomplete based on the number of foods reported.

Table 1 describes the demographic characteristics of the sample completing each of the recalls, as well as non-respondents. Among those completing the first recall, the mean age of the children was 8.7 years (SD 3.0); the age range was 4.1 to 15.3 years of age (IQR 5.7). Among those for whom a second recall was available, the mean age was 8.9 years (SD 2.9) with a range of 4.1 to 14.0 (IQR 4.3). The distribution of children’s ages was similar between those who did and did not have ASA24 recall data available; however, compared with non-respondents, parents completing ASA24 were more likely to have male children, have a single child, report a higher family income, and to be of European maternal ethnicity. Those completing ASA24 were also less likely to have children with BMI z scores > 2 (reflective of obesity).

**Table 1:** Demographic characteristics of samples completing first (N = 156) and second (N = 45) recalls, and of non-respondents (N = 301)

Characteristic	Non-respondents (N = 301)	First recall (N = 156)	Second recall (N = 45)
Age (years)^a^, mean (SD)	8.8 (2.9)	8.7 (3.0)	8.9 (2.9)
4–6 (n, %)	78 (25.9)	45 (28.8)	11 (24.4)
7–10 (n, %)	138 (45.8)	65 (41.7)	20 (44.4)
11–15 (n, %)	85 (28.2)	46 (29.5)	14 (31.1)
Sex^a^, n (%)			
Female	156 (51.8)	67 (43.0)	20 (44.4)
Male	145 (48.2)	89 (57.1)	25 (55.6)
Siblings^a^, n (%)			
0	30 (16.0)	21 (19.4)	7 (24.1)
1	115 (61.2)	64 (59.3)	16 (55.2)
2 or more	43 (22.9)	23 (21.3)	6 (20.7)
Missing^b^	113	48	16
Family income, n (%)			
Less than $30,000	3 (1.6)	2 (1.9)	1 (3.6)
$30,000 to $79,999	27 (14.8)	8 (7.5)	3 (10.7)
$80,000 to $149,999	49 (26.8)	38 (35.5)	10 (35.7)
$150,000 or more	104 (56.8)	59 (55.1)	14 (50.0)
Missing^b^	118	49	17
BMI z score^a^, n (%)			
≤ 1 (normal or underweight)	226 (75.6)	126 (81.8)	34 (75.6)
> 1 and ≤ 2 (overweight)	46 (15.4)	20 (13.0)	9 (20.0)
> 2 (obesity)	27 (9.0)	8 (5.2)	2 (4.4)
Missing	2	2	0
Maternal ethnicity, n (%)			
European	182 (67.9)	104 (73.2)	31 (77.5)
Asian	46 (17.2)	20 (14.1)	6 (15.0)
Other^c^	40 (14.9)	18 (12.7)	3 (7.5)
Missing	33	14	5

^a^Of the child (not the parent)

^b^Data on siblings and income were collected from the yearly age-specific questionnaires, which were not yet available for all participants.

^c^Includes Arab, African, Latin American, mixed ethnicity, and other

Table 2 describes the completion characteristics associated with the first recall and the second recall. The response rate for the first recall was 33.8% pre-incentive and 36.3% post-incentive. For the second recall, the response rate was 51.3% pre-incentive and 66.7% post-incentive. The median number of days between receiving an invitation to complete the first recall and completing it was 2.0 (IQR 7.5). The median number of days between completing the first recall and receiving an invitation for the second recall was 21.0 (IQR 13.0). For the second recall, the median time between receiving an invitation and completing was 7.0 days (IQR 7.0).

**Table 2:** Completion characteristics for the first (N = 156) and second (N = 45) recalls

Characteristics of completion	First recall (N = 156)	Second recall (N = 45)
Response rate overall, n (%)	163/471 (34.6)	46/78 (59.0)
Response rate pre-incentive, n (%)	105/311 (33.8)	20/39 (51.3)
Response rate post-incentive, n (%)	58/160 (36.3)	26/39 (66.7)
Number of attempts to complete, n (%)		
1	144 (92.3)	45 (100)
2	12 (7.7)	0 (0)
Recall indicative of normal intake, n (%)		
Much more than usual intake	4 (2.6)	0 (0)
Usual intake	144 (92.3)	43 (95.6)
Much less than usual intake	8 (5.1)	2 (4.4)
Time between receiving email invitation and completing recall (days), median (IQR)	2.0 (7.5)	7.0 (7.0)
Total recall session duration (min), median (IQR)	29.5 (17.0)	23.0 (14.0)
Number of foods reported, median (IQR)	18.0 (6.0)	18.0 (8.0)

The median number of foods reported was similar for the first and second recalls, at 18.0 (IQR 6.0) for the first recall and 18.0 (IQR 8.0) for the second. By comparison, the median number of foods reported on recalls collected within the 2015 CCHS was 14.0 (IQR 4.8); this difference may be partially attributed to differences in how multi-ingredient foods are coded in ASA24 versus CCHS. The median completion time for ASA24 was 29.5 (IQR 17.0) minutes for the first recall and 23.0 (IQR 14.0) minutes for the second. For both the first and second recalls, over 90% of respondents completed ASA24 in one attempt and indicated the foods reported for that day reflected their child’s usual intake. Recalls were collected from all days of the week although respondents were more likely to complete for weekdays (89% of first recalls and 91% of second recalls) compared with weekends (11% of first recalls and 9% of second recalls).

Table 3 shows the nutrient intake as estimated from the first and second ASA24 recalls. The values for all nutrients were similar between the first and second recalls. Further, when the TARGet Kids! first ASA24 recall was compared with national estimates among CCHS participants, the mean estimates were very similar with overlapping 95% CI for energy, protein, total fat and sodium, and only slightly higher for carbohydrates and total sugars and slightly lower for fiber. Based on the 95% CI, there was evidence of statistically significant differences between the first ASA24 recall and the CCHS for carbohydrates, fiber, and total sugars, but the ASA24 estimates were somewhat similar.

**Table 3:** Mean intake from the first (N = 156) and second (N = 45) recalls, compared with the CCHS^a^ (N = 4124)

Nutrient	Unit	First recall (N = 156)		Second recall (N = 45)		CCHS first recall (N = 4124)	
		Mean (95%CI)	Median (IQR)	Mean (95%CI)	Median (IQR)	Mean (95%CI)	Median (IQR)
Energy	(kcal/day)	1742.6 (1637.3–1847.9)	1704.0 (840.2)	1788.1 (1635.8–1940.4)	1740.3 (650.9)	1850.7 (1742.0–1959.4)	1728.0 (793.3)
Protein	(g/day)	70.5 (65.6–75.4)	65.0 (35.7)	75.1 (67.0–83.2)	74.3 (46.4)	70.6 (67.6–73.7)	64.0 (37.0)
Total fat	(g/day)	65.6 (60.2–71.0)	60.1 (39.4)	68.3 (59.6–77.0)	61.9 (39.1)	64.9 (58.9–70.9)	58.6 (36.7)
Carbohydrates	(g/day)	225.4 (211.6–239.2)	213.7 (98.5)	224.9 (204.8–245.0)	220.5 (100.2)	251.7 (240.1–263.3)	235.1 (111.5)
Fiber	(g/day)	18.3 (16.9–19.7)	17.1 (10.1)	17.4 (15.6–19.2)	16.5 (6.8)	15.6 (15.2–16.0)	14.1 (8.7)
Sodium	(mg/day)	2654.1 (2465.3–2842.9)	2498.7 (1472.0)	2709.8 (2434.7–2984.9)	2493.2 (1244.0)	2594.9 (2400.9–2788.8)	2357.9 (1393.5)
Total sugars	(g/day)	96.2 (89.0–103.4)	93.5 (54.6)	95.3 (85.3–105.3)	95.0 (53.4)	111.5 (103.8–119.1)	101.5 (63.0)
Added sugars	(g/day)	38.9 (34.2–43.6)	35.0 (31.9)	37.8 (30.3–45.3)	30.7 (28.3)	Not reported	Not reported

^a^Twenty-four-hour recall data collected within the 2015 CCHS (Canadian Community Health Survey) for children aged 4 to 15 years

## Discussion

This is one of the first studies to assess the feasibility of parent proxy-reported childhood dietary intake using ASA24. Overall, ASA24 appears to be a feasible approach for proxy-reported dietary assessment in children as young as 4 years of age. This finding aligns with that of Trolle and colleagues [34], who assessed the feasibility of parent proxy-reported in-person 24-h dietary recalls for a small group of 4–5-year-old children in Denmark and Spain. In that study, based on evaluation questionnaires provided after completion of the recalls, less than 15% of parents found it ‘difficult’ or ‘a little difficult’ to describe and report their child’s dietary intake; most found it ‘fairly easy’ or ‘easy’ [34]. The present study’s response rates, 34.6% for the first recall and 59.0% for the second, were similar to those of other studies in which adult members of an existing cohort were asked to complete additional dietary measures [35, 36]. Illner et al. [35] conducted dietary assessments with a random sample of five existing European cohorts, with an overall participation rate of 65.3% (participation rates for each cohort ranged from 37.5 to 87.5%). The response rate for the 2015 CCHS was also similar, at 61.6% [14]. Measuring the frequency of proxy under- and over-reporting of dietary intake was not possible in the current study. However, in a standardized daycare setting, parent proxy-reporting of the foods and beverages consumed by 2–5-year olds using ASA24 was found to have 80% close or exact matches when compared with true dietary intake as measured through direct observation [26]. Bӧrnhorst et al. [37] assessed the prevalence of misreporting for parent proxy-report of dietary intake using a European online 24-h recall for children ages 2–9 years. They found that the prevalence of under- and over-reporting of intake was 8.0% and 3.4%, respectively, when compared with Goldberg cutoff values for age-, sex-, and body size-specific basal metabolic rate [37].

Our study included children from 4 to 15 years of age (70% were ≤ 10 years of age), and parents were asked to complete the recall for their child. The literature suggests that proxy-reporting should be used for children below 10 years of age [25]. A validation study specific to the ASA24 suggested that children between the ages of 9 and 11 years may not be able to successfully complete the recall alone, and that more research is needed to determine the age at which parental assistance will no longer be required [20]. For these older children, it may be more appropriate to encourage parents to complete the ASA24 with their children (i.e., proxy-assisted responding). The CCHS administers proxy-reporting of dietary intake for children 1–5 years of age, with parent-assisted recalls for 6–12, and non-proxy (child completion) for those aged 12 years and up [33]. Although our study did not collect data on whether the child was present while their parent completed the recall, this information may have clarified the benefits of proxy-reporting compared with parent-assisted reporting among children of varying ages.

Systematic differences were observed between respondents and non-respondents on key characteristics, including child sex, number of siblings, family income level, maternal ethnicity, and BMI z score. These differences create the potential for selection bias, which can reduce generalizability and, in studies evaluating associations between diet and health outcomes, can lead to biased measures of association. This finding is consistent with previous research that has identified lack of completion of the ASA24 among low-income adults, as well as adults from certain ethnic groups [38, 39]; however, this has not been found in all studies [40]. Although we observed differences between the characteristics of respondents and non-respondents, estimated nutrient intakes were very similar to national estimates from the CCHS, which is a large, nationally representative survey. Overall, efforts are needed to increase response rates and support completion across demographic subgroups to enhance the generalizability of estimates yielded by cohorts, such as TARGet Kids!. For example, a systematic probability-based sampling technique could be used to improve generalizability. To increase response rate, the use of multiple methods to contact parents, including email, text messaging, and phone calls, may be beneficial.

This study offers valuable insights with regards to completion of new assessments by current members of cohorts. Demographic data were available on non-respondents through the TARGet Kids! cohort, enabling comparison characteristics of respondents versus non-respondents; many studies are not able to describe non-respondents. We were also able to evaluate changes in the response rate following introduction of an incentive due to a change in study protocol mid-study. Notably, survey participation improved minimally with the introduction of the incentive. This finding is in line with existing literature on survey response rates, which suggests that prepaid and cash-type incentives are more effective than promised and gift card-type incentives [41, 42].

However, several considerations are relevant to the interpretation of our findings. ASA24 was not designed for proxy-reporting and could not be customized to ask specifically about the foods and drinks that *“your child had”* rather than the foods and drinks that *“you had”.* Five recalls were excluded as it appeared, due to high amounts of caffeine or alcohol, the parents likely reported their own intake rather than their child’s. We cannot rule out the possibility that other parents may have inadvertently reported their own intake as well. A version of ASA24 that includes the option for proxy-reporting would broaden its applicability to studies with young children and other populations that may benefit from proxy-reporting. This study did not include any assessment of the usability of the ASA24 tool itself, hindering our understanding of parent’s experiences with completing ASA24 on behalf of their child and potential barriers to completion as well as strategies to overcome these barriers. Other studies have assessed the usability of ASA24 with measures such as the System Usability Scale [15]. Lastly, our study represents an urban population of children from Canada and may not be generalizeable to other populations.

## Conclusions

Many studies involving young children rely on dietary screening questionnaires or short FFQs, which tend to have higher levels of systematic error, or bias, compared with 24-h recalls [10, 11]. Overall, this study suggests ASA24 is a feasible approach for collecting recall data among young children, opening up possibilities for improved assessment of dietary intake in cohorts aiming to understand how dietary intake and other factors influence long-term health. However, further research should consider strategies to overcome barriers to completion among all cohort members.

## Supplementary Information

**Additional file 1.** CONSORT 2010 checklist of information to include when reporting a pilot or feasibility trial*.

**Additional file 2. **STROBE Statement—Checklist of items that should be included in reports of *cohort studies.*

## Data Availability

The data that support the findings of this study are available from TARGet Kids!, but restrictions apply to the availability of these data, which were used under license for the current study, and so are not publicly available. Data are however available upon request with approval of the TARGet Kids! Scientific Committee and institutional research ethics boards by contacting http://www.targetkids.ca/contact-us/. The CCHS data that were used in this study are available to authorized users through Statistics Canada.

## **Abbreviations**

FFQ: Food Frequency Questionnaire
ASA24: Automated Self-Administered 24-H Dietary Assessment Tool
CCHS: Canadian Community Health Survey
TARGet Kids!: The Applied Research Group for Kids
BMI: Body mass index
SD: Standard deviation
CI: Confidence interval
IQR: Interquartile range

## References

[1] World Cancer Research Fund/American Institute for Cancer Research. Recommendations and public health and policy implications. Continuous Update Project Expert Report. 2018. dietandcancerreport.org.

[2] Afshin A, Sur PJ, Fay KA, Cornaby L, Ferrara G, Salama JS, Mullany EC, Abate KH, Abbafati C, Abebe Z, Afarideh M, Aggarwal A, Agrawal S, Akinyemiju T, Alahdab F, Bacha U, Bachman VF, Badali H, Badawi A, Bensenor IM, Bernabe E, Biadgilign SKK, Biryukov SH, Cahill LE, Carrero JJ, Cercy KM, Dandona L, Dandona R, Dang AK, Degefa MG, el Sayed Zaki M, Esteghamati A, Esteghamati S, Fanzo J, Farinha CSS, Farvid MS, Farzadfar F, Feigin VL, Fernandes JC, Flor LS, Foigt NA, Forouzanfar MH, Ganji M, Geleijnse JM, Gillum RF, Goulart AC, Grosso G, Guessous I, Hamidi S, Hankey GJ, Harikrishnan S, Hassen HY, Hay SI, Hoang CL, Horino M, Islami F, Jackson MD, James SL, Johansson L, Jonas JB, Kasaeian A, Khader YS, Khalil IA, Khang YH, Kimokoti RW, Kokubo Y, Kumar GA, Lallukka T, Lopez AD, Lorkowski S, Lotufo PA, Lozano R, Malekzadeh R, März W, Meier T, Melaku YA, Mendoza W, Mensink GBM, Micha R, Miller TR, Mirarefin M, Mohan V, Mokdad AH, Mozaffarian D, Nagel G, Naghavi M, Nguyen CT, Nixon MR, Ong KL, Pereira DM, Poustchi H, Qorbani M, Rai RK, Razo-García C, Rehm CD, Rivera JA, Rodríguez-Ramírez S, Roshandel G, Roth GA, Sanabria J, Sánchez-Pimienta TG, Sartorius B, Schmidhuber J, Schutte AE, Sepanlou SG, Shin MJ, Sorensen RJD, Springmann M, Szponar L, Thorne-Lyman AL, Thrift AG, Touvier M, Tran BX, Tyrovolas S, Ukwaja KN, Ullah I, Uthman OA, Vaezghasemi M, Vasankari TJ, Vollset SE, Vos T, Vu GT, Vu LG, Weiderpass E, Werdecker A, Wijeratne T, Willett WC, Wu JH, Xu G, Yonemoto N, Yu C, Murray CJL (2019). Health effects of dietary risks in 195 countries, 1990–2017: a systematic analysis for the Global Burden of Disease Study 2017. The Lancet..

[3] Mikkilä V, Räsänen L, Raitakari OT, Pietinen P, Viikari J (2005). Consistent dietary patterns identified from childhood to adulthood: the cardiovascular risk in Young Finns Study. Br J Nutr..

[4] Movassagh EZ, Baxter-Jones ADG, Kontulainen S, Whiting SJ, Vatanparast H. Tracking dietary patterns over 20 years from childhood through adolescence into young adulthood: the Saskatchewan pediatric bone mineral accrual study. Nutrients. 2017;9(9). 10.3390/nu9090990 PMC5622750 28885565

[5] Kaikkonen JE, Mikkilä V, Magnussen CG, Juonala M, Viikari JSA, Raitakari OT (2013). Does childhood nutrition influence adult cardiovascular disease risk?—Insights from the Young Finns Study. Ann Med..

[6] Alam S, Lang JJ, Drucker AM, Gotay C, Kozloff N, Mate K, Patten SB, Orpana HM, Afshin A, Cahill LE (2019). Assessment of the burden of diseases and injuries attributable to risk factors in Canada from 1990 to 2016: an analysis of the Global Burden of Disease Study. CMAJ Open..

[7] Thompson FE, Kirkpatrick SI, Krebs-Smith SM, Reedy J, Schap TE, Subar AF (2015). The National Cancer Institute’s Dietary Assessment Primer: a resource for diet research. J Acad Nutr Diet..

[8] Subar AF, Freedman LS, Tooze JA, Kirkpatrick SI, Boushey C, Neuhouser ML, Thompson FE, Potischman N, Guenther PM, Tarasuk V, Reedy J, Krebs-Smith SM (2015). Addressing current criticism regarding the value of self-report Dietary Data12. J Nutr..

[9] Satija A, Yu E, Willett WC, Hu FB (2015). Understanding nutritional epidemiology and Its role in policy. Adv Nutr..

[10] Freedman LS, Commins JM, Moler JE, Arab L, Baer DJ, Kipnis V, Midthune D, Moshfegh AJ, Neuhouser ML, Prentice RL, Schatzkin A, Spiegelman D, Subar AF, Tinker LF, Willett W (2014). Pooled Results From 5 validation studies of dietary self-report Instruments using recovery biomarkers for energy and protein intake. Am J Epidemiol..

[11] Freedman LS, Commins JM, Moler JE, Willett W, Tinker LF, Subar AF, Spiegelman D, Rhodes D, Potischman N, Neuhouser ML, Moshfegh AJ, Kipnis V, Arab L, Prentice RL (2015). Pooled results from 5 validation studies of dietary self-r Instruments Using Recovery Biomarkers for Potassium and Sodium Intake. Am J Epidemiol..

[12] Thompson FE, Byers T (1994). Dietary Assessment Resource Manual. J Nutr.

[13] Subar AF, Kirkpatrick SI, Mittl B, Zimmerman TP, Thompson FE, Bingley C, Willis G, Islam NG, Baranowski T, McNutt S, Potischman N (2012). The Automated Self-Administered 24-Hour Dietary Recall (ASA24): a resource for researchers, clinicians, and educators from the National Cancer Institute. J Acad Nutr Diet..

[14] Health Canada: Reference Guide to Understanding and Using the Data: 2015 Canadian Community Health Survey—Nutrition. https://deslibris.ca/ID/10093153 (2017).

[15] Kirkpatrick SI, Gilsing AM, Hobin E, Solbak NM, Wallace A, Haines J, et al. Lessons from Studies to Evaluate an Online 24-Hour Recall for Use with Children and Adults in Canada. Nutrients. 2017;9(2). 10.3390/nu9020100 PMC5331531 28146125

[16] Kirkpatrick SI, Subar AF, Douglass D, Zimmerman TP, Thompson FE, Kahle LL, George SM, Dodd KW, Potischman N (2014). Performance of the Automated Self-Administered 24-hour Recall relative to a measure of true intakes and to an interviewer-administered 24-h recall123. Am J Clin Nutr..

[17] Thompson FE, Dixit-Joshi S, Potischman N, Dodd KW, Kirkpatrick SI, Kushi LH, Alexander GL, Coleman LA, Zimmerman TP, Sundaram ME, Clancy HA, Groesbeck M, Douglass D, George SM, Schap TRE, Subar AF (2015). Comparison of interviewer-administered and automated self-administered 24-hour dietary recalls in 3 diverse integrated health systems. Am J Epidemiol..

[18] Raffoul A, Hobin EP, Sacco JE, Lee KM, Haines J, Robson PJ, Dodd KW, Kirkpatrick SI (2019). School-age children can recall some foods and beverages consumed the Prior Day Using the Automated Self-Administered 24-Hour Dietary Assessment Tool (ASA24) without Assistance. J Nutr..

[19] Hughes AR, Summer SS, Ollberding NJ, Benken LA, Kalkwarf HJ (2017). Comparison of an interviewer-administered with an automated self-administered 24 h (ASA24) dietary recall in adolescents. Public Health Nutr..

[20] Diep CS, Hingle M, Chen T-A, Dadabhoy HR, Beltran A, Baranowski J, Subar AF, Baranowski T (2015). A validation study of the Automated Self-Administered 24-Hour Dietary Recall for Children (ASA24-Kids) among 9 to 11-year-old youth. J Acad Nutr Diet..

[21] Krehbiel CF, DuPaul GJ, Hoffman JA (2017). A validation study of the Automated Self-Administered 24-Hour Dietary Recall for Children, 2014 Version, at School Lunch. J Acad Nutr Diet..

[22] Baranowski T, Islam N, Baranowski J, Martin S, Beltran A, Dadabhoy H, Adame SH, Watson KB, Thompson D, Cullen KW, Subar AF (2012). Comparison of a web-based versus traditional diet recall among children. J Acad Nutr Diet..

[23] Smith AF, Baxter SD, Hitchcock DB, Finney CJ, Royer JA, Guinn CH (2016). Cognitive ability, social desirability, body mass index, and socioeconomic status as correlated of fourth-grade children’s dietary-reporting accuracy. Eur J Clin Nutr..

[24] Baxter SD, Smith AF, Litaker MS, Guinn CH, Shaffer NM, Baglio ML, Frye FHA (2004). Recency affects reporting accuracy of children’s dietary recalls. Ann Epidemiol..

[25] Livingstone MBE, Robson PJ, Wallace JMW (2004). Issues in dietary intake assessment of children and adolescents. Br J Nutr..

[26] Wallace A, Kirkpatrick SI, Darlington G, Haines J (2018). Accuracy of parental reporting of preschoolers’ dietary intake using an online self-administered 24-h Recall. Nutrients..

[27] Carsley S, Borkhoff CM, Maguire JL, Birken CS, Khovratovich M, McCrindle B, Macarthur C, Parkin PC, TARGet Kids! Collaboration. Cohort Profile: The Applied Research Group for Kids (TARGet Kids!). Int J Epidemiol. 2015;44(3):776–788, DOI: 10.1093/ije/dyu123. 10.1093/ije/dyu123 PMC4521122 24982016

[28] Hebert JR, Clemow L, Pbert L, Ockenet IS, Ockene JK (1995). Social desirability bias in dietary self-report may cmpromise the validity of dietary intake measures. Int J Epidemiol..

[29] Dodd KW, Guenther PM, Freedman LS, Subar AF, Kipnis V, Midthune D, Tooze JA, Krebs-Smith SM (2006). Statistical methods for estimating usual intake of nutrients and foods: a review of the theory. J Am Diet Assoc..

[30] Canadian Paediatric Society and Dieticians of Canada. Promoting optimal monitoring of child growth in Canada: using the new World Health Organization growth charts—Executive Summary. Paediatr Child Health. 2010;15(2):77–79. 10.1093/pch/15.2.77 PMC2865939 21286295

[31] Health Canada: Canadian Nutrient File Search Engine Online. https://food-nutrition.canada.ca/cnf-fce/index-eng.jsp (2018).

[32] National Cancer Institute: Reviewing and Cleaning ASA24® Data. https://epi.grants.cancer.gov/asa24/resources/asa24-data-cleaning.pdf (2018).

[33] Statistics Canada. CCHS 2015: Nutritional Component, General health and summary data for 24-hour dietary recall and nutritional supplements data set. ODESI. 2019. Identification number: cchs-82M0024-E-2015-nu-hs.

[34] Trolle E, Amiano P, Ege M, Bower E, Lioret S, Brants H (2011). Feasibility of repeated 24-h dietary recalls combined with a food-recording booklet, using EPIC-Soft, among preschoolers. Eur J Clin Nutr..

[35] Illner A-K, Harttig U, Tognon G, Palli D, Salvini S, Bower E, Amiano P, Kassik T, Metspalu A, Engeset D, Lund E, Ward H, Slimani N, Bergmann M, Wagner K, Boeing H (2011). Feasibility of innovative dietary assessment in epidemiological studies using the approach of combining different assessment instruments. Public Health Nutr..

[36] Galante J, Adamska L, Young A, Young H, Littlejohns TJ, Gallacher J, Allen N (2016). The acceptability of repeat Internet-based hybrid diet assessment of previous 24-h dietary intake: administration of the Oxford WebQ in UK Biobank. Br J Nutr..

[37] Börnhorst C, Huybrechts I, Ahrens W, Eiben G, Michels N, Pala V, Molnár D, Russo P, Barba G, Bel-Serrat S, Moreno LA, Papoutsou S, Veidebaum T, Loit HM, Lissner L, Pigeot I (2013). Prevalence and determinants of misreporting among European children in proxy-reported 24 h dietary recalls. Br J Nutr..

[38] Kupis J, Johnson S, Hallihan G, Olstad DL. Assessing the usability of the Automated Self-Administered Dietary Assessment Tool (ASA24) among low-income adults. Nutrients. 2019;11(1). 10.3390/nu11010132 PMC6357069 30634581

[39] Ettienne-Gittens R, Boushey CJ, Au D, Murphy SP, Lim U, Wilkens L (2013). Evaluating the feasibility of utilizing the Automated Self-Administered 24-hour (ASA24) dietary recall in a sample of multiethnic older adults. Procedia Food Sci..

[40] Kirkpatrick SI, Guenther PM, Douglass D, Zimmerman T, Kahle LL, Atoloye A, Marcinow M, Savoie-Roskos MR, Dodd KW, Durward C (2019). The provision of assistance does not substantially impact the accuracy of 24-hour dietary recalls completed using the Automated Self-Administered 24-H Dietary Assessment Tool among women with low incomes. J Nutr..

[41] Smith MG, Witte M, Rocha S, Basner M (2019). Effectiveness of incentives and follow-up on increasing survey response rates and participation in field studies. BMC Med Res Methodol..

[42] Mercer A, Caporaso A, Cantor D, Townsend R (2015). How much gets you how much? Monetary incentives and response rates in household surveys. Public Opinion Quarterly..

